# Ginsenoside Rg1 Prevents Cognitive Impairment and Hippocampal Neuronal Apoptosis in Experimental Vascular Dementia Mice by Promoting GPR30 Expression

**DOI:** 10.1155/2021/2412220

**Published:** 2021-12-03

**Authors:** Fengming Shen, Juan Wang, Feng Gao, Jingji Wang, Guoqi Zhu

**Affiliations:** ^1^Key Laboratory of Xin'an Medicine, Ministry of Education, Anhui University of Chinese Medicine, Hefei 230038, China; ^2^Department of Scientific Research, The Second Affiliation Hospital of Anhui University of Chinese Medicine, Hefei 230038, China

## Abstract

This study is aimed at investigating the potential roles of G protein-coupled estrogen receptor 1 (GPER, also known as GPR30) in the preventive effect of ginsenoside Rg1 against cognitive impairment and hippocampal cell apoptosis in experimental vascular dementia (VD) in mice. The effects of bilateral common carotid artery stenosis (BCAS) on GPR30 expression at mRNA level were evaluated. Thereafter, the BCAS mouse model was utilized to evaluate the protection of Rg1 (0.1, 1, 10 mg/kg, 14 days, *ip*). Spatial memory was evaluated by water Morris Maze 7 days post BCAS. After behavioral tests, neuronal apoptosis was detected by terminal deoxynucleotidyl transferase-mediated dUTP nick end labeling assay, and potential mechanisms were determined using western blotting and quantitative real-time PCR. Our results showed that GPR30 expression in the hippocampal region at mRNA level was promoted 30 min, 3 h, 6 h, and 24 h following BCAS. Ginsenoside Rg1 (1 or 10 mg/kg, 14 days, *ip*) promoted GPR30 expression in the hippocampus of model mice (after behavioral tests) but did not alter GPR30 expression in the hippocampus of control mice. Moreover, treatment of ginsenoside Rg1 (10 mg/kg) or G1 (5 *μ*g/kg), a GPR30 agonist, prevented BCAS-induced memory impairment and hippocampal neuronal loss and apoptosis and promoted the ratio of Bcl-2 to Bax expression in the hippocampus (after behavioral tests). On the contrary, G15 (185 *μ*g/kg), an antagonist of GPR30, aggravated BCAS-induced hippocampal neuronal loss and apoptosis. Finally, drug-target molecular docking pointed that Rg1 had a lower binding energy with GPR30 compared with Bax and Bcl-2. Together, our data implicate that ginsenoside Rg1 prevents cognitive impairment and hippocampal neuronal apoptosis in VD mice, likely through promoting GPR30 expression. These results would provide important implications for the application of Rg1 in the treatment of VD.

## 1. Introduction

Vascular dementia (VD) is a type of cognitive impairment syndrome caused by cerebral hypoperfusion or ischemic cerebrovascular diseases, which involves pathological changes in brain regions such as the hippocampus and cortex [1]. VD patients present with memory impairment, as well as behavioral and psychological symptoms such as anxiety and depression [2]. Presently, VD has become the second largest type of dementia threatening elderly people after Alzheimer's disease, accounting for about 15%-20% of dementia patients [3, 4].

*Panax ginseng* has been widely used to treat organic and psychosomatic diseases since ancient times [5]. Ginsenosides, especially ginsenoside Rg1, are considered the main active ingredients of ginseng [6]. Pharmacokinetic studies have shown that Rg1 can cross the blood-brain barrier and be widely distributed in the brain [7, 8]. As evidenced, Rg1 has a high efficacy in promoting neuroregeneration, improving neuroplasticity and immunity, etc. [9–11]. Especially, Rg1 has been extensively investigated in the treatment of dementia, such as improving cognitive dysfunction in mice with Alzheimer's disease [12]. Intriguingly, Rg1 was also reported to prevent cognitive impairment and neuronal damage in VD rats [13], but leaving the mechanisms largely open.

G protein-coupled estrogen receptor 1 (GPER, also known as GPR30) is a 7-transmembrane G protein-coupled receptor composed of 375 amino acids and has been identified as a new type of estrogen membrane receptor [14]. The activation of GPR30 is beneficial for neurological diseases and cardiovascular diseases [15–17]. GPR30 is involved in the treatment of a variety of brain diseases and mediation of various neurological functions, through regulating the release of neurotransmitters [18]. GPR30 activation could improve Parkinson's disease and cerebrovascular disease [19, 20] as well as ischemic cerebrovascular disease, likely via reducing neuronal death caused by ischemia, attenuating inflammatory response, etc. [21, 22]. In addition, GPR30 also plays an important role in fighting against aging [23, 24]. However, the potential function of GPR30 in VD is still not known.

Ginsenoside Rg1 has a steroid hormone-like steroid skeleton structure, which could activate the estrogen receptor through its characteristics of estrogen to exert the neuroprotective function [25]. Rg1 has been reported to regulate GPR30 to eliminate neuroinflammation in a Parkinson's disease model [26]. Bilateral common carotid artery stenosis (BCAS) was recognized for its mild reduction of cerebral blood flow and specific damage to white matter [27]. In this study, a mouse model of BCAS was utilized to investigate the potential roles of GPR30 in the protective effect of ginsenoside Rg1 on VD.

## 2. Materials and Methods

### 2.1. Animals and Drugs

Male C57BL/6J mice (3 months old, 20-25 g) were purchased from the Experimental Animal Center of Anhui Medical University (License number: SCXK (Wan) 2016-0009). The mice were kept in a room temperature of 22 ± 2°C and a relative humidity of 45-65% and experienced a 12 h light/dark cycle with food and water available *ad libitum*. All animal procedures were approved by the Ethics Committee of Anhui University of Chinese Medicine (Hefei, China).

Ginsenoside Rg1 was obtained from Yuanye Bio-Technology (HPLC ≥ 98%, Shanghai Yuanye Bio-Technology, China). GPR30 agonist (G1) and GPR30 inhibitor (G15) was purchased from Tocris (Bristol, UK). Rg1, G1, and G15 were dissolved in dimethyl sulfoxide (DMSO) and suspended in saline (DMSO concentration was lower than 0.1%), and the control group was injected with the same volume of normal saline.

### 2.2. Bilateral Common Carotid Artery Stenosis (BCAS) Mouse Model

The mice were anesthetized with 1% sodium pentobarbital (45 mg/kg). Through a midline cervical incision, both common carotid arteries (CCA) were exposed and freed from their sheaths. Two 4–0 silk sutures were placed around the distal and proximal parts of the right CCA. Then, the artery was gently lifted by these sutures and placed between the loops of the microcoil just below the carotid bifurcation. The microcoil was twined by rotating it around the CCA. 30 minutes later, another microcoil of the same size was twined around the left CCA. The rectal temperature was maintained between 36.5°C and 37.5°C. The cessation of cerebral blood flow (CBF) for >1 minute was avoided. The diameters of the CCAs were measured under direct inspection with an operation microscope (Model OMK2, Olympus Optical Co Ltd.) just before applying the microcoils. In the sham group, only bilateral CCA was exposed and isolated after anesthesia, and then, the wound was sutured. The death rate of the animals during BCAS was about 22%, which was consistent with previous publication [28]. There was no death in the sham group.

### 2.3. Experimental Groups

#### 2.3.1. Experimental Group 1

**Figure 1:**
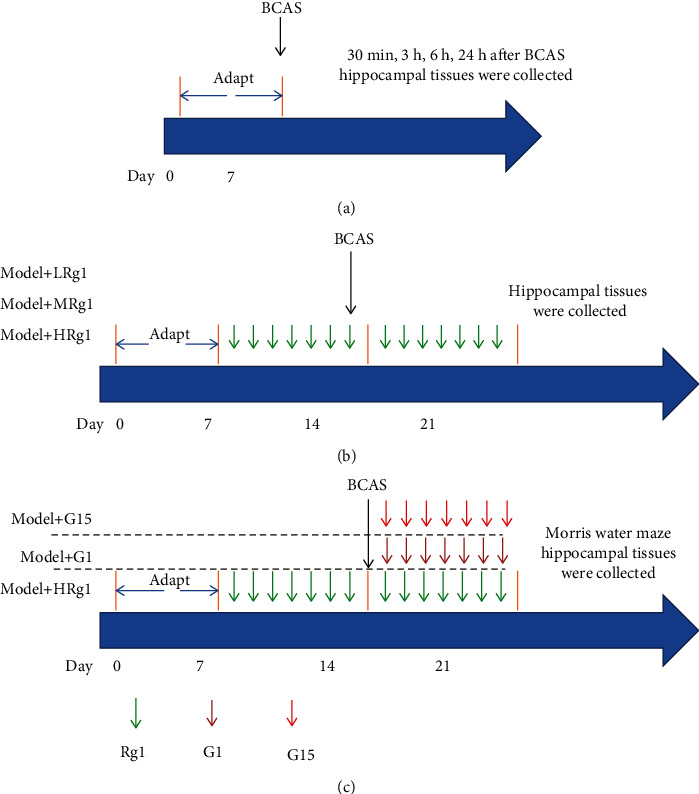
Schematic illustrates the study designs. (a) The mice were adapted for 7 days. Thereafter, BCAS was produced, and the hippocampal tissues were collected 30 min, 3 h, 6 h, and 24 h later. (b) The mice were adapted for 7 days. The mice in Rg1 groups received low, medium, and high doses of Rg1 for 14 consecutive days (one injection per day). Seven days after administration of Rg1, BCAS was produced in the mice. (c) The mice were adapted for 7 days. The mice in the Rg1 group received HRg1 for 14 consecutive days (one injection per day). Seven days after administration of Rg1, BCAS was produced in the mice. In the G1 and G15 groups, the mice received BCAS followed by G1 and G15, respectively. After treatments, Morris water maze was conducted. After the behavioral tests, the animals were decapitated following anesthesia with isoflurane (5%) and the hippocampi were collected.

The experiment was randomly divided into five groups (*N* = 5) (Figure 1(a)): a sham group and four model groups. The hippocampus was collected at four points of time after modeling (30 min, 3 h, 6 h, and 24 h) for the real-time PCR (Figure 1(a)).

#### 2.3.2. Experimental Group 2

The experiment was randomly divided into six groups (*N* = 5 (Figure 1(b)): a sham group, a model group, a LRg1 group (0.1 mg/kg), a MRg1 group (1 mg/kg), a HRg1 group (10 mg/kg) group, and a control+Rg1 (10 mg/kg) group. The animals in Rg1 groups were intraperitoneally administrated with Rg1 for 14 consecutive days (one injection per day). The doses of Rg1 were selected based on our previous publication [11]. The animals in other groups received similar volume of saline.

#### 2.3.3. Experimental Group 3

The experiment was randomly divided into five groups (*N* = 5) (Figure 1(c)): a sham group, a model group, a model+Rg1 (10 mg/kg) group, a GPR30 agonist (G1)+model group, and a GPR30 inhibitor (G15)+model group. The animals in the model+Rg1 group were intraperitoneally administrated with Rg1 one injection per day for 14 consecutive days. In the G1+model and G15+model groups, the mice were administrated with one injection of G1 (5 *μ*g/kg subcutaneously (sc)) and G15 (185 *μ*g/kg, sc) per day for 7 consecutive days after modeling. The doses of G1 and G15 were selected by referring a previous publication [29].

### 2.4. Morris Water Maze (MWM)

MWM was performed one week after surgery to assess the behavioral performance between the different groups as previously described [30]. On day 22 after drug treatment, the rats began the 6-day MWM test with 5-day training consisting of visible platform trials as well as spatial probe trials on the sixth day. Animals were allowed to explore the platform within 90 s and kept on the platform for 10 s. If the mouse did not reach the platform within 90 s, the training was terminated with the animal gently directed to the platform by hand for 30 s. A probe test was conducted on day 6. After behavioral tests, the animals were decapitated following anesthesia with isoflurane (5%) and the hippocampi were collected.

### 2.5. Quantitative Real-Time PCR (qPCR)

30 min, 3 h, 6 h, and 24 h after BCAS, the mice were anesthetized with isoflurane (5%) and decapitated. Thereafter, the hippocampi were isolated on ice. Total RNA was extracted with Trizol reagent (Takara Bio Inc.) and reversely transcription into cDNA using reverse transcription kit (Beyotime Institute of Biotechnology, China). Cycle parameters were as follows: initial denaturation at 95°C for 10 min, followed by 40 cycles of sequential incubations at 95°C for 15 s and at 60°C for 1 min. After the end of the cycle, the data was analyzed using the instrument's supporting software to obtain the cycle threshold (Ct value). GPR30 expression was normalized to glyceraldehyde-3-phosphatedehydrogenase (GADPH) and calculated by the 2^-∆∆CT^ method as previously described [31]. The primers were listed as follows: GPR30 (forward 5′-TCATTTCTGCCATGCACCCA-3′ and reverse 5′-GTGGACA-GGGTGTCTGATGT-3′) and GAPDH (forward 5′-AACTTTGGCATTGTGGAAGG-3′ and reverse 5′-ACACATTGGGGGTAGGAACA-3′).

### 2.6. Western Blotting

The hippocampal tissues were added to protein lysis solution (RIPA: PMSF = 100: 1) and homogenized for lysis to detect the expression of Bax, Bcl-2, and GPR30. Protein samples were separated by sodium dodecyl sulfate polyacrylamide gel electrophoresis (12% gel) for 50 min at 120 V and transferred onto a nitrocellulose membrane for 2 h at 200 mA. The membranes were blocked with PBS containing 0.1% Tween-20 (PBST) and 5% fat-free milk for 2 h at room temperature. After blocking, the membrane was incubated with primary antibody against Bax (1 : 1000, Cell Signaling Technology (CST), Danvers, MA, USA), Bcl-2 (1 : 1000, CST, Danvers, MA, USA), and GPR30 (1 : 1000, CST, Danvers, MA, USA) at 4°C overnight. After being washed three times with PBST (3 × 10 min), the membranes were incubated with horseradish peroxidase-conjugated goat anti-rabbit IgG (1 : 10,000, Zs-bio) 2 h at room temperature. Finally, the membrane was developed with a chemiluminescent solution (ECL, Thermo Fisher Technology, USA). ImageJ software was used to analyze the optical density of the bands.

### 2.7. Immunohistochemical Staining

The mouse brain was fixed in 4% paraformaldehyde for 24 h, cryoprotected in 30% sucrose for 24 h at 4°C, and sectioned on a freezing microtome at 20 *μ*m. Sections were blocked in 0.1 M phosphate-buffered saline (PBS) containing 10% goat serum and 0.4% TritonX-100 for 1 h and then incubated with a primary antibody against NeuN (1 : 400, CST, Danvers, MA, USA). Composition of the primary antibody dilution included PBS containing 0.3% Triton X-100 and 1% bovine serum albumin (BSA). The sections were washed 3 times in PBS (15 min each) and then incubated with goat anti-rabbit IgG H&L (Alexa Fluor® 488) (1 : 200, Life Technologies, Carlsbad, CA, USA) for 2 h at room temperature. The morphology of neurons in the hippocampus was observed under a microscope (Olympus, Tokyo, Japan). ImageJ software was used to calculate the number of NeuN^+^ cells.

### 2.8. TUNEL Staining

The mouse brain was fixed in 4% paraformaldehyde for 24 h, cryoprotected in 30% sucrose for 24 h at 4°C, and sectioned on a freezing microtome at 20 *μ*m. TUNEL stained was carried out according to the instructions of the TUNEL apoptosis detection kit (Beyotime Institute of Biotechnology, China). After staining, images were taken with an FV1000 Olympus confocal laser scanning microscope (Olympus, Tokyo, Japan). ImageJ software was used to analyze the image and calculate the number of apoptotic cells.

### 2.9. Drug-Target Molecular Docking

To verify the binding energy of ginsenoside Rg1 to target proteins, we monitored molecular docking between ginsenoside Rg1 and GPR30 (PDB ID 4X13), Bax (PDB ID 4S0O), and Bcl-2 (PDB ID 4CIM), respectively. We downloaded the 3D PDB format files of GPR30, BAX, and Bcl-2 from the PDB database and the 2D SDF of active ingredients from the PubChem database and imported the 2D structure into ChemBio3D14.0 software for structural optimization and revealed the 3D mol2 structure. Then, we imported the drug molecules and proteins into the AutoDock Tool for hydrogenation, charge calculation, atom addition, and ROOT docking. The algorithm was Local Search Parameters, and the lower the binding energy indicated, the better the docking result. We used Pymol software to analyze the docking result.

### 2.10. Statistical Analysis

Data were presented as the mean ± standard error of mean (SEM) and analyzed with GraphPad Prism 5.0 software (GraphPad Inc., San Diego, CA, USA). One-way ANOVA followed by Bonferroni's test was applied to compare between-group differences. A value of *P* < 0.05 was considered significant.

## 3. Results

### 3.1. Effects of BCAS on GPR30 mRNA Expression in the Hippocampus

**Figure 2:**
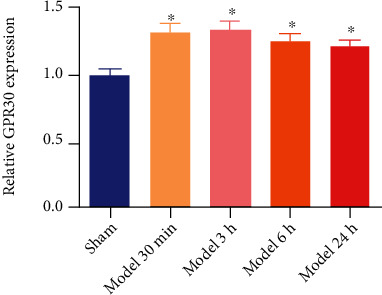
BCAS modeling promoted the expression of hippocampal GPR30. GPR30 mRNA expression was promoted 30 min, 3 h, 6 h, and 24 h in the hippocampus after BCAS. ^∗^*P* < 0.05, vs. sham (*N* = 5 in each group).

The expression of GPR30 mRNA at 30 min, 3 h, 6 h, and 24 h after BCAS was detected. 30 min, 3 h, 6 h, and 24 h after establishment of BCAS, GPR30 expression at mRNA level in the hippocampus was higher than that in the sham group (Figure 2, *P* < 0.05), which suggested that the expression of GPR30 increased after the modeling of BCAS.

### 3.2. Effects of Ginsenoside Rg1 on the Expression of GPR30 in the Hippocampus

**Figure 3:**
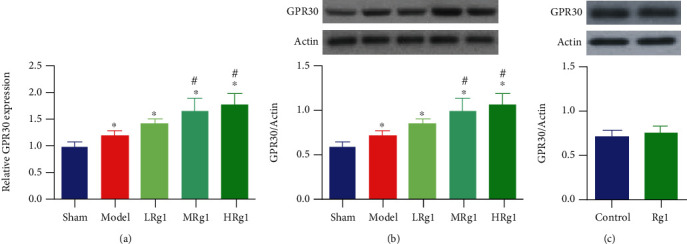
Ginsenoside Rg1 promoted the hippocampal GPR30 expression in the BCAS model animals but had no effect in normal animals. (a) GPR30 mRNA expression; (b) GPR30 protein expression in the hippocampus of mice receiving BCAS and ginsenoside Rg1; (c) GPR30 protein expression in the hippocampus of normal mice receiving ginsenoside Rg1. ^∗^*P* < 0.05, vs. sham; ^#^*P* < 0.05, vs. the model group (*N* = 5 in each group).

The results of qPCR and western blot suggested that the expression of GPR30 at both mRNA and protein levels in the hippocampus in the model group were significantly higher than those in the sham group (Figures 3(a) and 3(b)). Compared with the model group, there was no significant difference in the expression of GPR30 in the hippocampus of mice in the low-dose group of Rg1, while the expression of GPR30 at mRNA and protein levels in the hippocampus of high and medium doses of ginsenoside Rg1 groups were upregulated significantly (Figures 3(a) and 3(b), *P* < 0.05). By contrast, in normal animals, treatment with ginsenoside Rg1 did not promote GPR30 expression in the hippocampus (Figure 3(c), *P* > 0.05).

### 3.3. Ginsenoside Rg1 Improves Memory in BCAS Model Mice

**Figure 4:**
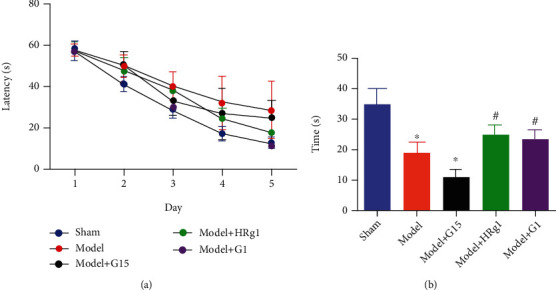
Ginsenoside Rg1 improves memory in BCAS model animals. (a) The latency to find the platform during five-day training. (b) Time spent in target quadrant. ^∗^*P* < 0.05, vs. sham; ^#^*P* < 0.05, vs. the model group (*N* = 5 in each group).

We also tested spatial memory after Rg1 treatment. The learning trends among groups were not altered during training (Figure 4(a)). However, memory retrieval represented by time spent in the platform quadrant was significantly prolonged in G1-treated and Rg1-treated mice, which was significantly decreased in G15-treated mice (Figure 4(b), vs. model, *P* < 0.05). These results suggested that G1 and Rg1 improved memory in BCAS model mice.

### 3.4. Effects of Ginsenoside Rg1 on Hippocampal Neuronal Loss Caused by BCAS Modeling

**Figure 5:**
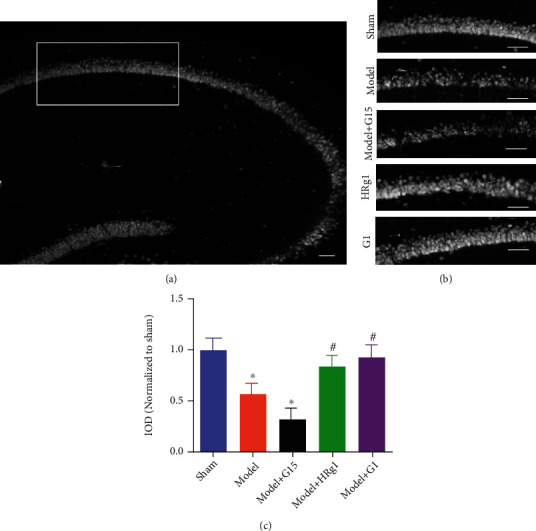
Ginsenoside Rg1 inhibited BCAS-induced loss of hippocampal neurons. (a) Observation area; (b) representative images of each group; scale bar: 50 *μ*m; (c) NeuN^+^ cell counts. ^∗^*P* < 0.05, vs. the sham group, ^#^*P* < 0.05, vs. the model group (*N* = 5 in each group).

As the expression of GPR30 was most significantly increased in the high-dose ginsenoside group, the high-dose ginsenoside was selected in subsequent experiments. Additionally, GPR30 agonist (G1) and antagonist (G15) were selected to verify the functions of GPR30. Immunohistochemical staining results showed that BCAS modeling stimulated the loss of hippocampal neurons compared with the sham group (Figures 5(a)-5(c)), while ginsenoside Rg1 or G1 treatment prevented BCAS-induced neuronal loss. By contrast, G15 further aggravated the injury of BCAS (Figure 5(c), vs. the model, *P* < 0.05).

### 3.5. Effects of Ginsenoside Rg1 on Hippocampal Neuronal Apoptosis and Expression of Apoptosis-Related Proteins in BCAS Mouse Model

**Figure 6:**
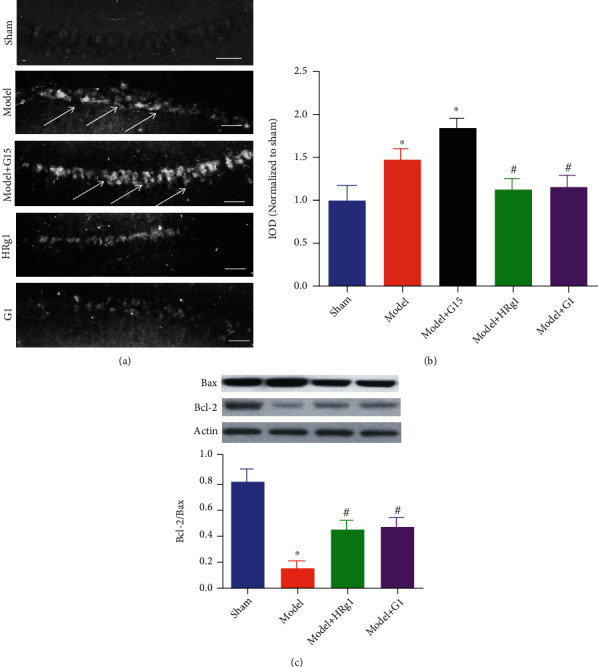
Ginsenoside Rg1 inhibited hippocampal neuronal apoptosis induced by BCAS modeling. (a) TUNEL staining; arrows indicate the apoptotic cells; scale bar: 50 *μ*m. (b) Apoptotic cell count; (c) Bcl-2/Bax expression level and representative bands of Bcl-2 and Bax. ^∗^*P* < 0.05, vs. the sham group, ^#^*P* < 0.05, vs. the model group (*N* = 5 in each group).

TUNEL staining showed that BCAS modeling caused apoptosis of hippocampal neurons compared with sham group, while ginsenoside Rg1 and G1 treatment improved neuronal apoptosis induced by BCAS modeling (Figure 6(a)). The apoptotic cell count in the model group increased significantly compared with the sham group. Compared with the model group, the hippocampal apoptotic cell count in the high-dose of ginsenoside group and G1 group decreased significantly (Figure 6(b), *P* < 0.05). G15 further aggravated the apoptosis caused by BCAS modeling (Figure 6(b), vs. the model, *P* < 0.05).

Western blot results showed that the ratio of hippocampal Bcl-2/Bax of the model group mice decreased significantly compared with the sham group. Compared with the model group, the ratio of hippocampal Bcl-2/Bax of the Rg1 administration group and G1 group increased significantly (Figure 6(c), *P* < 0.05).

### 3.6. Molecular Docking Pattern and Results

**Figure 7:**
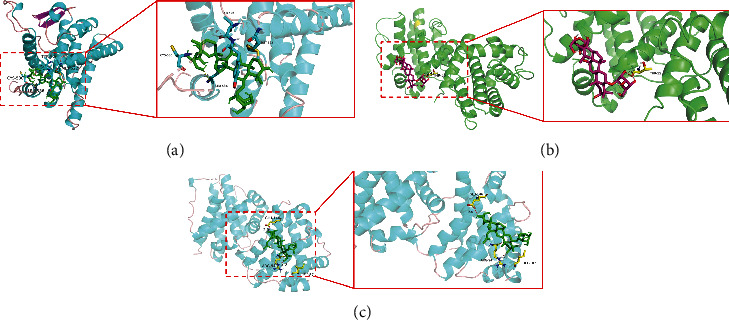
Molecular docking pattern and results. (a) Molecular docking results of Rg1 and GPR30; (b) molecular docking results of Rg1 and Bax; (c) molecular docking results of Rg1 and Bcl-2. Binding energy between Rg1 and GPR30, Bcl-2, or Bax was -4.69, -0.53, and -0.17 kcal/mol.

As ginsenoside Rg1 influenced GPR30, Bax, and Bcl-2 expression, we further investigated the possible relationships of Rg1 with GPR30, Bax, and Bcl-2. Molecular docking results showed that Rg1 had a lower binding energy with GPR30 than with Bax and Bcl-2 (Figure 7). Binding energy between Rg1 and GPR30, Bcl-2, or Bax was -4.69, -0.53, and -0.17 kcal/mol, respectively.

## 4. Discussion

Our findings indicated that Rg1 improved memory in BCAS model mice. Notably, Rg1 increased the GPR30 level and ameliorated hippocampal neuronal loss and apoptosis. These data provided critical evidence supporting the protective effects of Rg1 on VD.

Ginseng has been used for thousands of years to treat aging and memory disorders [32]. *Panax ginseng* extract attenuates neuronal injury and cognitive deficits in VD rats [33]. Ginsenosides, the main active ingredients of ginseng, play an important role in the central nervous system (CNS) disorders and widely act on cell membrane receptors [34]. Several studies have demonstrated that ginsenoside Rg1 could improve cognitive function in chemotherapy-induced cognitive impairment, repeated alcohol exposure-induced cognitive deficits, and Alzheimer's disease [12, 35, 36]. Moreover, ginsenoside Rg1 has ameliorative effects on various brain injuries, such as focal cerebral ischemia injury, diabetic complicated cerebral infarction, and ischemic-reperfusion brain injury [37–39]. Thus, it is of great significance to investigate the effect of Rg1 on VD. However, there are few researches on its protective mechanism against VD, and its mechanism is unclear, although possible mechanisms include apoptosis, oxidative stress, and inflammation [13].

In the CNS, estrogen has typical beneficial effects for trophic nerve, antioxidant, anti-inflammatory, and antiapoptosis [40]. A previous study has demonstrated that estrogen could also increase the expression of prosurvival factor Bcl-2 in hippocampal neurons [41]. The nutritional nerve function of estrogen can protect nerve growth factor adequately and reduce the damage of neurons [42]. Estrogen exerts its biological effects by mediating estrogen membrane receptors [43]. As a new type of estrogen membrane receptor, the activation of GPR30 may have a vital function in biological process. Activation of GPR30 after myocardial ischemia can protect against myocardial cell injury and play a protective role by maintaining mitochondrial function [44]. The upregulation of GPR30 modulates the increased expression of Bcl-2, thereby preventing mitochondrial-dependent apoptosis, which may be achieved by activating the PI3K/Akt pathway [45, 46]. The expression of GPR30 is affected by the level of serum estrogen [47], to exclude this factor, male animals or ovariectomized (OVX) female animals are generally used in estrogen-related studies. GPR30 expression was increased after ischemia-reperfusion in OVX female mice [22]. Here, we selected male mice and the results showed that BCAS modeling promoted the expression of GPR30 in the hippocampus of male mice, suggesting that cerebral ischemia induced by BCAS may activate GPR30 to a certain extent and play a neuroprotective role.

Ginsenoside Rg1 has estrogen-like activity and may exert estrogenic effects via rapid activation of membrane-associated ER and GPR30 [48]. Our studies have demonstrated that ginsenoside treatment promoted the expression of GPR30 in the hippocampus of BCAS modeling mice but had no effect on the expression of GPR30 in normal animals. In addition, molecular docking results showed that Rg1 had a lower binding energy with GPR30. This further suggested that ginsenoside Rg1 may activate GPR30 through estrogen-like properties. We treated animals with the GPR30 agonist and GPR30 inhibitor, indicating that GPR30 agonist treatment improved BCAS-induced neuronal loss and apoptosis, while GPR30 inhibitor aggravated BCAS-induced neuronal loss and apoptosis. Recent studies have found that the expression of GPR30 is not only mediated by estrogen, but the regulation of GPR30 by microRNA is also an important signaling pathway [49]. Therefore, a further study of the upstream regulatory pathway of GPR30 and the specific mechanism of the association between ginsenoside Rg1 and GPR30 is warranted.

The hippocampus is an essential area in the brain associated with learning and memory [50–53]. It has been suggested that the hippocampus is the most important region of transient cerebral ischemia for brain injury, which is consistent with the pathological characteristics of patients with global cerebral ischemia caused by cardiac arrest [54, 55]. Our study demonstrated that Rg1 pretreatment can inhibit the BCAS-induced hippocampal neuronal loss, indicating that Rg1 can improve hippocampal neuronal damage caused by cerebral ischemia. Neuronal damage caused by cerebral ischemia includes apoptosis and necrosis [52]. In the early stage of cerebral ischemia, the main mode of neuronal death is apoptosis [56]. There are many pathways that regulate apoptosis, and they interfere and regulate each other at different levels. The *Bcl-2* gene family plays a decisive role in the process of apoptosis. It includes the antiapoptotic factor Bcl-2 and the proapoptotic factor Bax. Bcl-2 and Bax function as a dimer, and the ratio between the two determines the apoptosis [57]. Our results indicated that Rg1 pretreatment can inhibit BCAS-induced hippocampal neuronal apoptosis. We selected Bcl-2 and Bax to investigate apoptosis-related pathways. The data suggested that ginsenoside Rg1 reversed the decrease of the ratio of Bcl-2 and Bax in the hippocampus caused by BCAS modeling, further confirming the protective effect of Rg1 on hippocampal neuronal apoptosis. There are various ways in cell death, and Rg1 alleviates cell death by influencing other death pathways, such as necrosis and pyroptosis [58, 59]. Additionally, neuroinflammation regulated by microglia M1 polarization and NLRP3 inflammasome pathway activation may also contribute to the cerebral injury [30, 60, 61]. Rg1 may also alleviate VD by influencing these death pathways, which needs further investigation.

In conclusion, ginsenoside Rg1 improves hippocampal neuronal injury in BCAS model mice, and GPR30 plays a pivotal role in vascular dementia. Our study would provide important implication for the application of Rg1 or the Chinese medicine with ginsenoside Rg1 as the major component in the treatment of VD.

## Data Availability

The data used to support the findings of this study are included within the article.
